# Nutrition and Autoimmune Diseases

**DOI:** 10.3390/nu17132176

**Published:** 2025-06-30

**Authors:** Rosaria Maddalena Ruggeri, Silvana Hrelia, Maria Cristina Barbalace

**Affiliations:** 1 Endocrinology Unit, Department of Human Pathology of Adulthood and Childhood DETEV “G. Barresi”, University of Messina, 98125 Messina, Italy; rmruggeri@unime.it; 2 Department for Life Quality Studies, Alma Mater Studiorum, University of Bologna, 47921 Rimini, Italy; silvana.hrelia@unibo.it

Autoimmune diseases (AIDs) represent a growing challenge in global health, quietly expanding their impact across populations and burdening healthcare systems with complex, chronic conditions. Rooted in a breakdown of immunological tolerance, AIDs occur when the immune system mistakenly targets the body’s own tissues, leading to inflammation, tissue damage, and often irreversible functional impairment [1]. While the causes of AIDs remain elusive, the global rise in their incidence—affecting approximately 4% of the population, disproportionately among women—suggests a pressing need to better understand the interplay of the factors involved.

Genetics provides a framework, but environmental influences have taken center stage in recent decades, particularly in industrialized nations. Among these, nutritional factors are increasingly seen not just as contributors to health, but as potential modulators of autoimmune responses [2]. This evolving recognition marks a pivotal shift in our approach to autoimmunity, bringing nutrition from the periphery of clinical consideration to the forefront of immunological research.

Nutrition is far more than a matter of calories or macro and micronutrients—it is a dynamic, complex player in immune health. It shapes physical barriers like the skin and gut lining, determines the balance and function of the gut microbiome, and modulates the innate and adaptive arms of the immune system. The way we nourish ourselves affects how macrophages respond to threats, how T and B cells recognize self from non-self, and how effectively the body maintains immunological balance [3].

This intricate relationship between nutrition and immunity is bidirectional. Just as diet influences immune responses, so immune processes affect nutritional metabolism and dietary needs [4]. Inflammatory responses can alter appetite, nutrient absorption, and metabolic demand, further complicating the clinical picture of AIDs.

Given this complexity, it is no surprise that researchers are focusing on nutrition not only to understand the causes of autoimmune conditions but also to explore innovative therapeutic avenues. From the potential of anti-inflammatory dietary patterns to targeted use of micronutrients and nutraceutical bioactive compounds, nutritional strategies could offer accessible, cost-effective ways to prevent or manage AIDs.

Diet significantly impacts autoimmune disorders, especially, but not only, those affecting the thyroid, such as Hashimoto’s thyroiditis. The Mediterranean diet, rich in anti-inflammatory foods like olive oil, fish, fruits, vegetables, and whole grains, has been shown to support thyroid health by reducing systemic inflammation and improving immune function. Studies suggest that the diet’s high content of antioxidants, omega-3 fatty acids, and selenium may play a protective role in autoimmune thyroid conditions by modulating immune responses and reducing oxidative stress, which is often a key factor in thyroid dysfunction [5,6].

This Special Issue was addressed to collect rigorous investigations into these connections—ranging from preclinical studies to clinical trials and meta-analyses. We seek to illuminate how specific nutrients, dietary habits, and metabolic pathways intersect with immune regulation, autoimmunity, and patient outcomes. By bridging the gap between nutrition science and immunology, we hope to uncover new strategies for managing autoimmune diseases and improving the lives of those affected.

The Special Issue includes two reviews and four original articles that delve into recent research aimed at describing the role of dietary habits and functional compounds in modulating AIDs like autoimmune thyroiditis, autoimmune encephalomyelitis, multiple sclerosis, rheumatic immune diseases, and sepsis.

Osowiecka et al. (Contribution 1) utilized a cross-sectional study to investigate the relationship between diet quality, nutritional status, and quality of life in 147 women with Hashimoto’s thyroiditis (HAT). Using standardized tools (pHDI-10 for diet, ThyPROpl for quality of life, and GSRS for gastrointestinal symptoms), researchers found that 80% of participants had a low-quality diet, with no significant differences in nutritional status or quality of life between low and medium diet quality groups. Despite the lack of a direct link between diet quality and well-being in this sample, most women exhibited poor eating habits, elevated BMI, and reduced quality of life, particularly in areas such as fatigue, depression, and anxiety. The study underscores the need for further interventional research and the development of a HAT-specific dietary assessment tool.

On the other hand, the review of Barbalace et al. (Contribution 2) highlights the Mediterranean diet (MD) and its components—such as omega-3 PUFAs, polyphenols, and fiber—for their potential in modulating immune responses. The authors examine the MD beneficial effects across both systemic (e.g., rheumatoid arthritis, lupus) and organ-specific (e.g., Hashimoto’s thyroiditis) autoimmune conditions. The findings support the MD as a promising complementary strategy to improve outcomes in autoimmune disorders.

The relationship between iodine status and thyroid autoimmunity (TAI) in Chinese children and adolescents after two decades of universal salt iodization was examined in Contribution 3. Despite generally adequate iodine levels, thyroid autoimmunity—marked by the presence of TPOAb and TgAb—remains a concern due to its impact on growth and development. TAI was associated with factors like age, sex, and urban–rural residence. While no strong overall link between iodine levels and TAI was found, certain subgroups showed specific risks: low iodine-to-creatinine ratios increased TgAb positivity in males, and high BMI and iodine levels were risk factors for subclinical hypothyroidism in antibody-negative individuals. The study underlies the need for personalized iodine strategies based on individual thyroid antibody status.

The study of Herrada et al. (Contribution 4) investigated the effects of yerba mate (YM), a traditional Latin American infusion, on multiple sclerosis (MS) using a model of mouse experimental autoimmune encephalomyelitis (EAE). Mice treated with YM showed reduced MS-like symptoms, decreased immune cell infiltration into the central nervous system, and less demyelination compared to controls. YM also increased the population and suppressive function of regulatory T cells, which help control inflammation. These findings suggest that YM may promote an immunosuppressive environment and could serve as a cost-effective therapeutic option.

MS is a growing autoimmune disease among young adults; it has unclear causes but is influenced by both genetic and environmental factors. Diet’s role in MS risk remains poorly understood due to limited large-scale studies. Utilizing the UK Biobank, Barbero Mazzucca et al. (Contribution 5) examined the relationship between dietary and lifestyle habits and the development of MS. The study found that moderate oily fish consumption and weekly alcohol intake may offer protective effects. Additionally, a non-significant but favorable trend was observed with adherence to the MD. These findings highlight the potential influence of diet on MS risk and the need for further in-depth research in this area for MS.

Finally, the interesting review of Gabrielli et al. (Contribution 6) focused on sepsis, a severe and often fatal response to infection, that remains a major concern in hospitals, particularly outside intensive care units, where nutritional management is less defined. This narrative review explores the metabolic disruptions caused by sepsis, approaches to nutritional assessment, and targeted supplementation strategies, including nutrient-specific interventions and gut microbiota modulation. By consolidating current evidence, the review aims to inform effective nutritional care for septic patients in non-intensive settings and identifies key areas needing further investigation.

Understanding the impact of dietary habits on autoimmune disorders is increasingly vital, as these conditions are rising globally and often lack curative treatments. While genetic and environmental factors are known contributors, diet represents a modifiable risk factor with the potential to influence immune function, inflammation, and gut microbiota—key elements in autoimmune disease development and progression. Investigating dietary patterns, such as adherence to anti-inflammatory diets (e.g., the Mediterranean diet) or specific nutrient intake, could offer insights into prevention strategies, symptom management, and improved quality of life for affected individuals. Yet, despite its potential, diet remains an underexplored area in autoimmune research, emphasizing the urgent need for large-scale, evidence-based studies.

As the burden of AIDs continues to rise, future perspectives reflect the need for integrated, multidisciplinary approaches. Nutrition, long overlooked in immunological discourse, may yet prove to be one of our most potent tools in the fight against autoimmune disease.
